# Feeding capability in the extinct giant *Siamogale melilutra* and comparative mandibular biomechanics of living Lutrinae

**DOI:** 10.1038/s41598-017-15391-9

**Published:** 2017-11-09

**Authors:** Z. Jack Tseng, Denise F. Su, Xiaoming Wang, Stuart C. White, Xueping Ji

**Affiliations:** 1 Department of Pathology and Anatomical Sciences, Jacobs School of Medicine and Biomedical Sciences, University at Buffalo, Buffalo, New York, 14214 USA; 2 Division of Paleontology, American Museum of Natural History, Central Park West at 79th Street, New York, New York 10024 USA; 3 Department of Vertebrate Paleontology, Natural History Museum of Los Angeles County, 900 Exposition Boulevard, Los Angeles, California, 90007 USA; 4 Department of Paleobotany and Paleoecology, Cleveland Museum of Natural History, 1 Wade Oval Drive, University Circle, Cleveland, Ohio, 44106 USA; 5 Key Laboratory of Vertebrate Evolution and Human Origins of Chinese Academy of Sciences, Institute of Vertebrate Paleontology and Paleoanthropology, Chinese Academy of Sciences, Beijing, 100044 China; 6 School of Dentistry, University of California, Los Angeles, 10833 Le Conte Ave., Los Angeles, California, 90095 USA; 7 Yunnan Institute of Cultural Relics and Archaeology, 15-1, Chunmingli, Chunyuan Xiaoqu, Kunming, Yunnan 650118 China

## Abstract

At 50 kg in estimated weight, the extinct *Siamogale melilutra* is larger than all living otters, and ranks among the largest fossil otters. The biomechanical capability of *S*. *melilutra* jaws as related to their large size is unknown but crucial to reconstructing the species’ potentially unique ecological niche. Here we compare the mandibular biomechanics of *S*. *melilutra* using engineering-based performance measures against ten extant otter biomechanical models. Despite a wide range of feeding preferences from durophagy to piscivory, living otter species exhibit a linear relationship between mandible stiffness and volume, as expected in isometric model scaling. In contrast, *S*. *melilutra* models exhibit a six-fold increase in stiffness from expected stiffness-volume relationships calculated from extant species models. Unlike stiffness, mechanical efficiency of biting is conserved among living otters and in *S*. *melilutra*. These findings indicate that although similar to living bunodont otters in morphology and biting efficiency, jaw strength in *S*. *melilutra* far surpasses molluscivores such as sea otters and Cape clawless otters, even after accounting for size. Therefore, *Siamogale* represents a feeding ecomorphology with no living analog, and its giant size and high mandibular strength confer shell-crushing capability matched only by other extinct molluscivores such as the marine bear *Kolponomos*.

## Introduction

Otters (Lutrinae) are a group of mustelid carnivorans that has evolved cranial and post-cranial adaptations to living and hunting in aquatic environments worldwide^1^. The thirteen living otter species are distributed throughout the Americas, Europe, Asia, and Africa, and are found in both freshwater and marine habitats. The diets of living otters are diverse, ranging from piscivorous specialists, omnivores, to molluscivorous durophages (Table 1, Supplementary Table S1). The fossil records of living otter genera are fragmentary, possibly because of relatively rapid divergence from the late Miocene onward^2^. As such, the macroevolutionary patterns of ecomorphological diversity in otters are incompletely known.

**Table 1:** Otter species analyzed in the study.

Genus	Species	Biogeography	Feeding Ecomorphology	Specimen
*Aonyx*	*capensis*	Africa	Durophage	CMNH 17620
*Aonyx*	*cinerea*	SE Asia	Durophage	AMNH 101638
*Enhydra*	*lutris*	N. Pacific Coast	Durophage	AMNH 24186
*Hydrictis*	*maculicollis*	Central Africa	Omnivore	AMNH 84807
*Lontra*	*canadensis*	North America	Omnivore	AMNH 254476
*Lontra*	*felina*	S. Pacific Coast	Fish specialist	AMNH 48193
*Lontra*	*longicaudis*	Mexico, S. America	Fish specialist	AMNH 98589
*Lutra*	*lutra*	Eurasia	Omnivore	AMNH 206592
*Lutrogale*	*perspicillata*	S. Asia	Fish specialist	AMNH 204747
*Pteronura*	*brasiliensis*	S. America	Fish specialist	AMNH 98594
*Siamogale*	*melilutra*	E. Asia	This Study	IVPP V 23271

Feeding ecomorphology classification is based on natural history observations listed in Supplementary Table S1. A species is considered (1) a durophage if the principal food item observed are crabs or other hard-shelled invertebrates, (2) omnivore if no single type of food is preferred over others, and (3) fish specialist if the principal food item is identified as any fish species.

**Figure 1:**
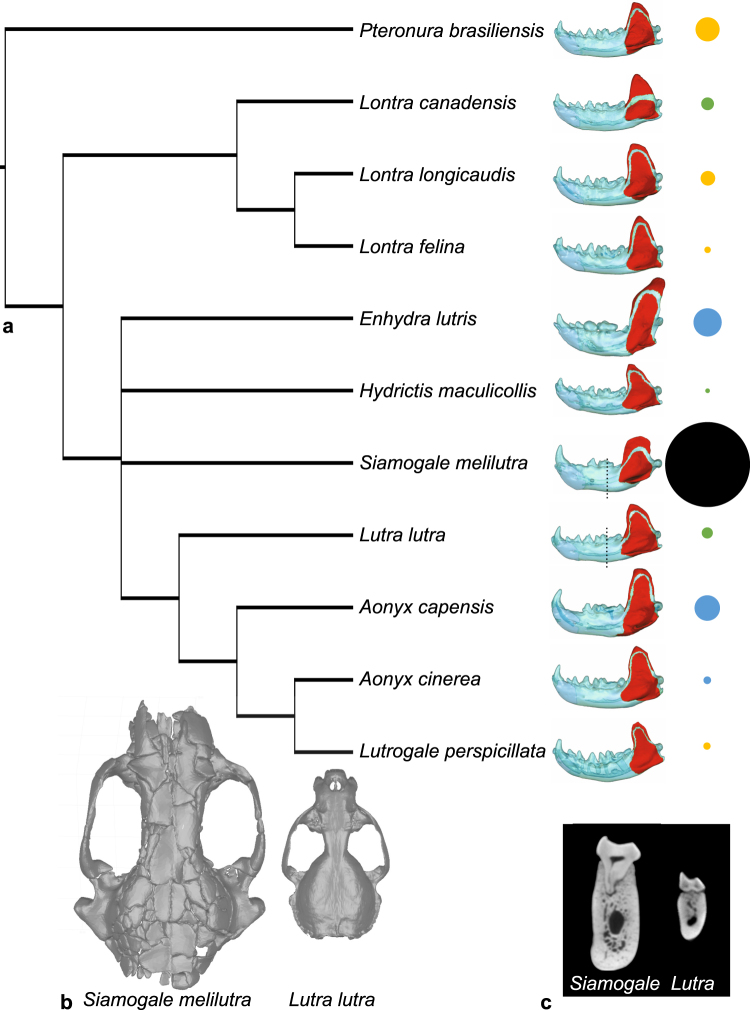
(**a**) Phylogeny of otter species analyzed in the study. Relationships are based on the work of Koepfli *et al*.^2^ and Wang *et al*.^3^ Left lower jaws with temporalis and masseter attachment sites labeled in red are shown for each species. Jaws are scaled to the same length for visual comparison. Sizes of circles indicate relative jaw model volume; orange color denotes fish specialists, green denotes omnivores, and blue denotes durophages. The feeding preference of *Siamogale* is discussed in this study. (**b**) Comparison of crania of *Siamogale melilutra* and *Lutra lutra*. (**c)**. Coronal sections from CT scans of *S*. *melilutra* and *L*. *lutra* taken at the m1 trigonid/talonid boundary.

As a case in point, although living otters range in body mass from ~4 kg in the spotted-necked otter to almost 45 kg in the sea otter, the largest otter species are extinct. Among them, the recently described *Siamogale melilutra* from southeast and east Asia has been estimated to weigh at least 50 kg^3^. *Siamogale* is also considered one of the largest otters by linear skull and jaw dimensions. Using engineering-based performance measures from biomechanical model simulations, we examined the mandibular feeding capability of *Siamogale melilutra* relative to ten living otter species (Fig. 1) based on mandibular specimen IVPP V 23271 recovered from Shuitangba (Yunnan Province, China)^3^. Based on its overall robust morphology, bunodont dentition, and large size, we hypothesize that *Siamogale*’s mandible behaved biomechanically much like those of the living durophages *Enhydra lutris* (sea otter) and *Aonyx capensis* (Cape clawless otter) compared to non-durophagous otters in having 1) functionally differentiated posterior (crushing) and anterior (prey capturing) teeth, 2) higher mechanical efficiency (ratio between input muscle forces and output bite forces) and lower strain energy (a measure of structural stiffness; higher energy meaning lower stiffness), and 3) overall lower stress levels throughout the mandible during biting.

We tested these hypotheses using comparative finite element analysis, utilizing a tested protocol^4^ that incorporates the complex geometry of biological structures (in this case, the mandible), model uncertainty generated during the modeling process, and user-input values of material properties, constraints, and loads into approximations of the deformations and displacements in bony structures in simulated musculoskeletal function scenarios. We analyzed mechanical efficiency, strain energy, and von Mises stress distributions from these simulations to test our hypotheses of increased functional differentiation, efficiency, and stiffness in durophages and *Siamogale* relative to other otter species.

## Results

**Figure 2:**
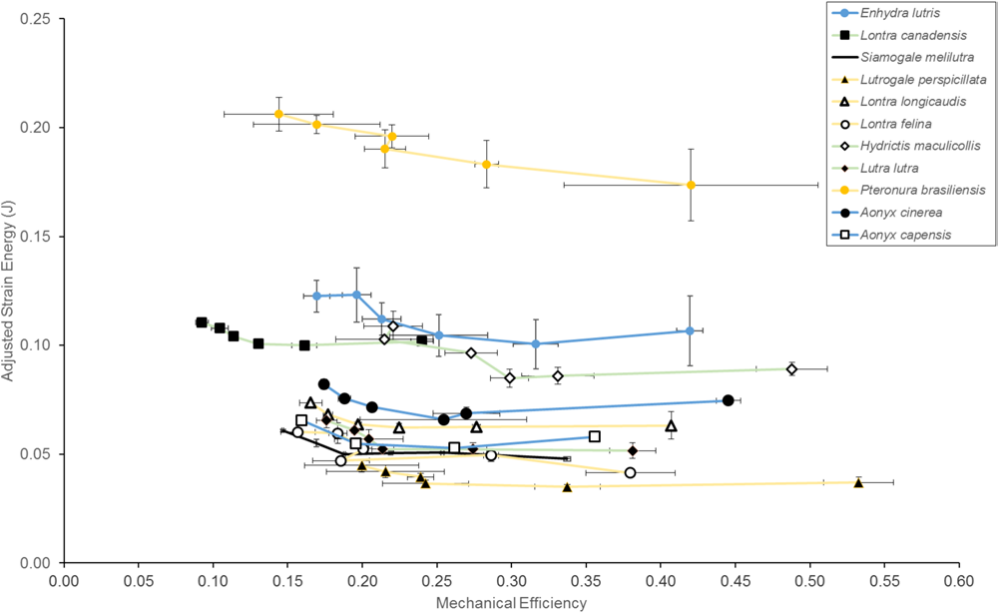
Plot of mechanical efficiency (ME) to adjusted strain energy (SE) across the tooth loci tested in each otter species. The curve for each species represents canine to second molar bite positions (from left to right); for *Aonyx*, only canine, p4, and m1-2 were present. Error bars indicate 95% confidence interval for the ME and SE values, calculated from the low, medium, and high resolution models constructed for each species. Colors of the curves indicate piscivores (orange), omnivores (green), and molluscivores (blue).

The mechanical efficiency (ME) values of all tooth loci simulated in all species range from 0.09 to 0.53 (Fig. 2, Table 2). There is extensive overlap among the species curves; based on overlapping 95% confidence intervals of the tooth loci ME values, no two species are significantly different in their ME ranges. The adjusted strain energy (SE) values range from 0.04 to 0.21; most of the species are clustered within the 0.04 to 0.13 range. *Pteronura brasiliensis*, the giant river otter, shows significantly higher SE (lower stiffness) than all other species analyzed. *Siamogale* has relatively low SE values (third lowest), only higher than *Lutrogale perspicillata* and *Lontra felina* (Fig. 2). Dietary categories do not correspond to consistent ME or SE differences (Fig. 2).

**Table 2:** Mean mechanical efficiency (ME) and adjusted strain energy (SE) outputs from the finite element simulations.

Taxon		cME	p2ME	p3ME	p4ME	m1ME	m2ME
*Aonyx capensis*	mean	0.1591	—	—	0.1957	0.2618	0.3559
	95%CI	0.0064	—	—	0.0073	0.0164	0.0101
*Aonyx cinerea*	mean	0.1744	0.1880	0.2066	0.2545	0.2697	0.4454
	95%CI	0.0025	0.0042	0.0027	0.0557	0.0223	0.0082
*Enhydra lutris*	mean	0.1694	0.1963	0.2131	0.2513	0.3163	0.4196
	95%CI	0.0088	0.0097	0.0129	0.0329	0.0151	0.0087
*Hydrictis maculicollis*	mean	0.2208	0.2148	0.2729	0.2987	0.3311	0.4880
	95%CI	0.0196	0.0327	0.0175	0.0127	0.0243	0.0238
*Lontra canadensis*	mean	0.0926	0.1045	0.1139	0.1304	0.1611	0.2401
	95%CI	0.0043	0.0057	0.0019	0.0016	0.0083	0.0077
*Lontra felina*	mean	0.1568	0.1836	0.2008	0.1858	0.2864	0.3796
	95%CI	0.0114	0.0060	0.0028	0.0191	0.0050	0.0299
*Lontra longicaudis*	mean	0.1655	0.1771	0.1972	0.2247	0.2767	0.4071
	95%CI	0.0074	0.0031	0.0024	0.0009	0.0027	0.0009
*Lutra lutra*	mean	0.1761	0.1947	0.2045	0.2138	0.2743	0.3811
	95%CI	0.0069	0.0020	0.0230	0.0072	0.0093	0.0155
*Lutrogale perspicillata*	mean	0.1996	0.2155	0.2391	0.2426	0.3374	0.5327
	95%CI	0.0381	0.0394	0.0086	0.0288	0.0220	0.0233
*Pteronura brasiliensis*	mean	0.1442	0.1695	0.2199	0.2152	0.2833	0.4203
	95%CI	0.0366	0.0424	0.0247	0.0137	0.0079	0.0849
*Siamogale melilutra*	mean	0.1469	0.1693	0.1881	0.2191	0.2536	0.3373
	95%CI	0.0006	0.0003	0.0006	0.0009	0.0011	0.0024
		**cSE**	**p2SE**	**p3SE**	**p4SE**	**m1SE**	**m2SE**
*Aonyx capensis*	mean	0.0656	—	—	0.0548	0.0528	0.0579
	95%CI	0.0021	—	—	0.0017	0.0009	0.0030
*Aonyx cinerea*	mean	0.0821	0.0756	0.0716	0.0659	0.0687	0.0746
	95%CI	0.0023	0.0011	0.0009	0.0011	0.0030	0.0011
*Enhydra lutris*	mean	0.1226	0.1232	0.1120	0.1046	0.1005	0.1067
	95%CI	0.0073	0.0125	0.0075	0.0096	0.0113	0.0160
*Hydrictis maculicollis*	mean	0.1088	0.1027	0.0966	0.0849	0.0859	0.0891
	95%CI	0.0069	0.0021	0.0013	0.0041	0.0039	0.0031
*Lontra canadensis*	mean	0.1105	0.1078	0.1041	0.1007	0.0999	0.1018
	95%CI	0.0011	0.0009	0.0009	0.0009	0.0009	0.0020
*Lontra felina*	mean	0.0602	0.0596	0.0522	0.0470	0.0495	0.0414
	95%CI	0.0018	0.0047	0.0014	0.0011	0.0029	0.0014
*Lontra longicaudis*	mean	0.0736	0.0681	0.0637	0.0621	0.0624	0.0632
	95%CI	0.0004	0.0004	0.0008	0.0021	0.0011	0.0064
*Lutra lutra*	mean	0.0654	0.0609	0.0569	0.0522	0.0522	0.0516
	95%CI	0.0032	0.0008	0.0042	0.0003	0.0029	0.0036
*Lutrogale perspicillata*	mean	0.0448	0.0419	0.0395	0.0365	0.0350	0.0369
	95%CI	0.0030	0.0029	0.0016	0.0014	0.0011	0.0024
*Pteronura brasiliensis*	mean	0.2062	0.2014	0.1960	0.1902	0.1832	0.1736
	95%CI	0.0078	0.0041	0.0053	0.0088	0.0109	0.0164
*Siamogale melilutra*	mean	0.0609	0.0551	0.0500	0.0505	0.0506	0.0478
	95%CI	0.0006	0.0017	0.0005	0.0009	0.0005	0.0010

95% confidence intervals (CI) were calculated from simulation results of low, medium, and high resolution models analyzed for each species. Abbreviations: c, canine; p2-4, premolars 2 to 4; m1-2, molars 1 to 2. For raw data outputs see Supplementary Tables S4–S6.

**Figure 3:**
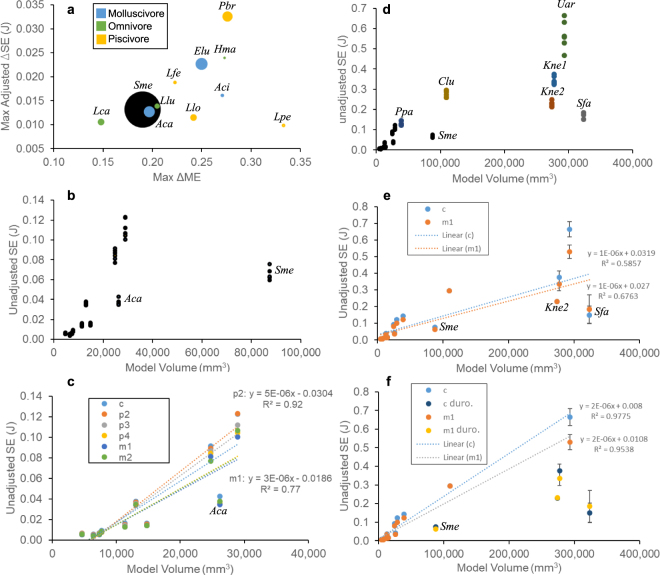
Plots of functional differentiation and size scaling relationships. (**a**) functional differentiation as measured by maximum differences in ME and adjusted SE (corrected for input force and volume differences) values across the tooth loci of each species; sizes of circles indicate relative jaw model volumes. (**b**) Model volume plotted against unadjusted SE values for all otter species analyzed. (**c**) Linear regression analysis of volume to SE values in living otter species only; equations are shown for the fitted lines that bracket the other tooth loci regression lines. Gray lines indicate 95% confidence intervals for regression line. (**d**) Unadjusted SE versus model volume for otters and six additional species jaw models. (**e**) Linear regression analysis using all seventeen jaw models, comparing the canine and m1 tooth loci, respectively. (**f**) Linear regression analysis excluding the fossil outliers. Abbreviations: Aci, *Aonyx cinerea*; Aca, *Aonyx capensis*; Clu, *Canis lupus;* Elu, *Enhydra lutris*; Hma, *Hydrictis maculicollis*; Kne1, *Kolponomos newportensis* with tall coronoid reconstruction; Kne2, *Kolponomos newportensis* with low coronoid reconstruction; Lca, *Lontra canadensis*; Lfe, *Lontra feline*; Llo, *Lontra longicaudis*; Llu, *Lutra*; Lpe, *Lutrogale perspicillata*; Pbr, *Pteronura brasiliensis*; Ppa, *Panthera pardus*; Sfa, *Smilodon fatalis*; Sme, *Siamogale melilutra*; Uar, *ursus arctos*.

Functional differentiation of the dentition, measured by plotting the maximum differences in ME and SE across the tooth loci simulations for each species (larger maximum differences indicate higher degree of functional differentiation across the tooth row), does not show any separation of dietary categories (Fig. 3a). However, two species most differentiated in either ME or SE values are both piscivores - *Pteronura brasiliensis* is most differentiated in SE values, whereas *Lutrogale perspicillata* is most differentiated in ME values. *Siamogale* is low in both ME and SE differentiation, its values higher only than those of *Lontra canadensis*. *Lontra canadensis*, an omnivore, shows the least differentiated ME and SE values among the species analyzed. When the unadjusted SE values are plotted against model volumes, *Siamogale* exhibits low strain energy (high stiffness) values relative to its mandibular model volume (Fig. 3b). A series of linear regression analyses of the SE to volume relationship among living otter species indicate a significant linear relationship across all tooth loci simulations (Fig. 3c, Table 3). Given the regression equations, the SE values in *Siamogale* is more than six times lower than expected given the relationship observed in living otter species. Within living otter species, the molluscivore *Aonyx capensis* shows the largest departure from the linear regression line and has a stiffer mandible for its volume than all other living otters. The linear relationships between SE values and model volume remain significant (*p* < 0.01) when phylogenetic relationships are taken into account in phylogenetic generalized least squares regression analyses.

**Table 3:** Linear regression analysis statistics.

	Extant otters				Extant carnivorans			
	Intercept	Vol Coef	*p*	R^2^	Intercept	Vol Coef	*p*	R^2^
c SE	−0.0224	0.000004	0.001	77.89	0.0081	0.000002	<0.001	97.75
p2 SE	−0.0304	0.000005	<0.001	92.00	0.0060	0.000002	<0.001	98.40
p3 SE	−0.0281	0.000005	<0.001	92.32	0.0125	0.000002	<0.001	96.76
p4 SE	−0.0200	0.000004	0.001	76.90	0.0092	0.000002	<0.001	96.58
m1 SE	−0.0186	0.000003	0.001	76.74	0.0108	0.000002	<0.001	95.38
m2 SE	−0.0196	0.000003	0.001	78.16	0.0112	0.000002	<0.001	94.59

Results for linear regression analysis between model volume (Vol) and raw strain energy values (SE) for each tooth position (c, canine; p, premolar, m, molar) listed include: intercept, coefficient for the volume variable, *p*-values for the volume variable coefficient (no intercept values are significant at the *p* = 0.05 level), and R^2^ (goodness of fit). Two sets of regression analyses were conducted; one for extant otters, the other with combined extant otter and additional carnivoran data from literature.

When non-otter carnivoran species models are included in the SE-volume plot (Fig. 3d), the SE values of *Siamogale* remain below the fitted regression line both with (Fig. 3e) and without (Fig. 3f) the three fossil outliers (*Siamogale*, *Kolponomos* [a marine bear], and *Smilodon* [sabertoothed felid]). All three fossil outliers have significantly lower SE values (no overlap between fitted regression line and 95% confidence intervals of simulated values) than predicted by the linear regression model using thirteen extant carnivoran species.

**Figure 4:**
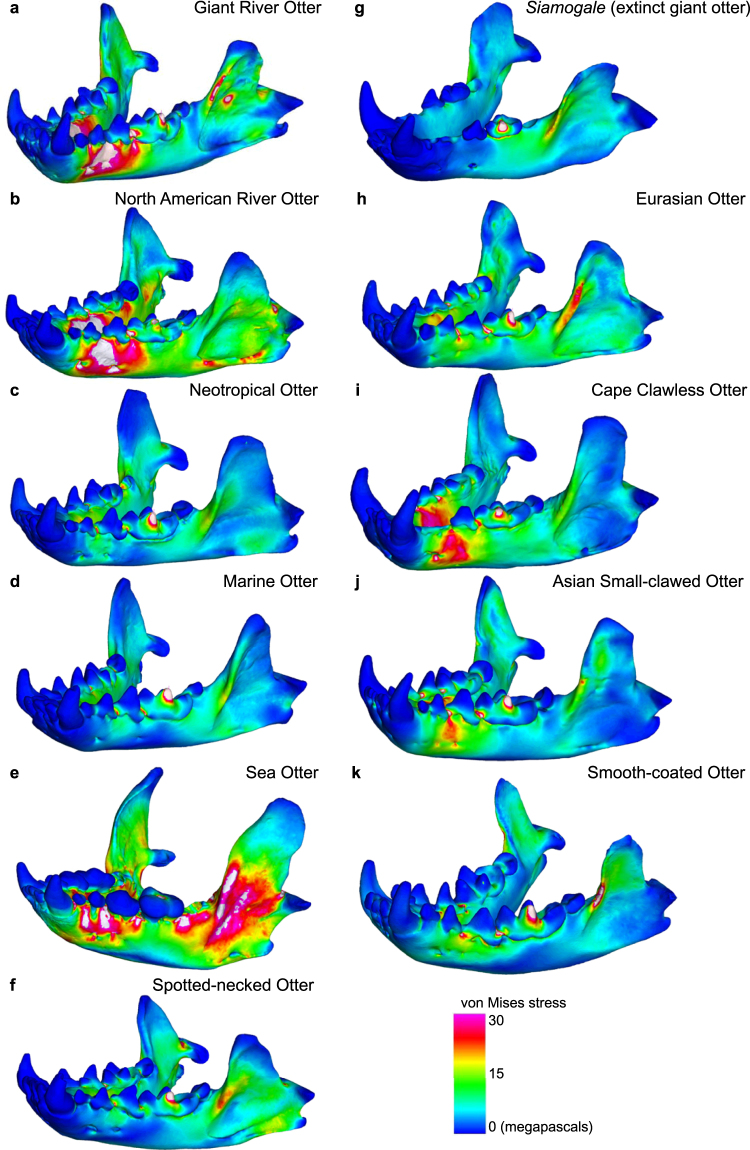
. von Mises stress distributions in ‘first molar bite’ finite element simulations. Results shown are for left unilateral m1 bites. (**a**) *Pteronura brasiliensis*. (**b**) *Lontra canadensis*. (**c**) *Lontra longicaudis*. (**d**) *Lontra felina*. (**e**) *Enhydra lutris*. (**f**) *Hydrictis maculicollis*. (**g**) *Siamogale melilutra*. (**h**) *Lutra lutra*. (**I**) *Aonyx capensis*. (**j**) *Aonyx cinerea*. (**k**) *Lutrogale perspicillata*. Warmer colors indicate higher stress, cooler colors, lower stress. White shapes indicate von Mises stress values beyond the preset scale of 0–30 MPa (megapascals).

In von Mises stress distributions, the mandibles of five otter species (*Pteronura brasiliensis*, *Lontra canadensis*, *Enhydra lutris*, *Aonyx capensis*, *Aonyx cinerea*) are visibly more highly stressed than the other species (Fig. 4a,b,e,i,j). All otters exhibit heightened stress levels at the coronoid process (sites of temporalis and masseter muscle attachments) and below the bite position, but only living otters exhibit an elevated region of stress at the ramus between the p3-p4 teeth. *Siamogale* exhibits low levels of stress in the mandible anterior to the bite position and a small region of elevated stress at the anterior ridge of the coronoid process (Fig. 4g).

## Discussion

Results of the finite element simulations provided no support for increased functional differentiation (Fig. 3a), mechanical efficiency (Fig. 2), stiffness (Fig. 2), or consistent differences in stress distributions (Fig. 4) in living durophagous otter species compared to non-durophagous species. Therefore, our hypotheses of similarity between *Siamogale* and living durophagous otters, as well as predicted differences between living durophagous and non-durophagous otter species, are not supported by the data. In fact, the relationship between the unadjusted SE values and model volumes of living otter species is significantly linear (Fig. 3c, Table 3). This stiffness-volume linearity is a relationship expected from isometric scaling of a given finite element model with input forces set proportional to surface area^5^. In Dumont *et al*.’s derivation of proper scaling coefficients for maintaining proportional output values (i.e., isometric model scaling), a linear relationship is expected between strain energy and model volume given that input force to area ratio is held constant. This linear relationship is expected for models of identical geometry at different volumes, but such relationship is not necessarily a null expectation for a comparative species sample of varying geometry and varying volumes, such as the dataset analyzed in this study. All our models were assigned input forces proportional to the muscle attachment areas (highlighted on the mandible in Fig. 1), and simulations were conducted using original volumes of the jaw models. Given these modeling parameters, a significant linear relationship among the ten living otter species analyzed indicates that total strain energy-volume scaling relationships follow isometric model scaling principles regardless of the actual differences in dietary preference or mandibular morphology.

This strain energy-volume linearity is an unexpected result from a functional perspective, as living otter species exhibit a wide range of diets that differ significantly in prey material properties (from soft tissues to hard exoskeletons) and in hunting strategy (from snapping bites when capturing fish, to crushing bites when breaking hard-shell invertebrates)^6–13^. In addition, some of the general anatomical gradients previously identified in non-pinniped durophagous carnivorans compared to non-durophagous carnivorans are reflected in mandible shapes of molluscivorous lutrines^14^. For example, taller and broader coronoid processes and deeper mandibular corpi in the molluscivores *Enhydra* and *A*. *capensis* (Fig. 1a). A significantly linear strain energy-volume relationship suggests that such morphological differences do not relate to mandible stiffness (as measured by strain energy) differences. Nevertheless, the scatter around the linear regression lines indicates that small departures in mandible stiffness from linearity (which assumes identical geometry) could still be associated with biomechanical differences associated with mandible shape differences (although; as an example, *Aonyx capensis*, a crab-eater that has not been observed to use shell-breaking tools (i.e., hard-shelled prey items are processed orally), does exhibit a relatively stiff mandible model profile (Fig. 2) as well as lower than expected SE values given its model volume (Fig. 3b,c; values falling below the 95% CI). Along the same lines, the high stiffness values of the *Siamogale* models could be related to its robust mandibular corpus, which has thick cortical walls that increase the second moment of area and therefore resistance to bending (Fig. 1c)^15^.

In light of the linear relationship between SE and volume among the ten living otter species examined, the simulation outputs of *Siamogale* shows a remarkable departure from the typical otter pattern (Fig. 3b), and suggests that living otters are poor analogs for understanding the biomechanical adaptations of *Siamogale*. When the SE-volume relationship is analyzed in the broader context of other carnivoran species including omnivore (brown bear), hypercarnivore (wolf), and fossil species inferred to have diets involving high jaw loads (the extinct marine bear *Kolponomos*, and the extinct sabertooth *Smilodon*), the linear relationship appears to hold among the living species (Fig. 3f). The inferred molluscivore *Kolponomos* is roughly equidistant from the fitted regression line in vertical distance as *Siamogale*, indicating similar degrees of departure from the living carnivoran pattern, and a significant decrease in SE (or an increase in stiffness) relative to their jaw volumes. *Kolponomos* has previously been shown, using finite element simulations, to possess stiff mandibles with deep mandibular symphysis, both being structural characteristics consistent with a shell-prying hunting strategy followed by oral crushing of hard-shelled invertebrates^16^. The mandibular symphysis of *Siamogale* appears relatively deepened compared to living otters (Fig. 1), but to a lesser extent than *Kolponomos*. Furthermore, the deepening of the mandibular ramus at the m1-m2 location, rather than at the location of the symphysis, indicates that *Siamogale* was not adapted to prying hard-shelled invertebrates with the incisor tooth row to the extent inferred in *Kolponomos*. However, both *Siamogale* and *Kolponomos* have mandibular morphology (robust and deep rami) and biomechanics (high jaw stiffness relative to volume) consistent with extensive use of the jaw as a crushing tool.

The living otter species that is most significantly below the linear regression line is *Aonyx capensis*, an oral crusher without observed tool-using ability for breaking shells^6^. Compared to *Aonyx*, the tool-using sea otter *Enhydra lutris* has a mandible with a level of stiffness expected from isometric model scaling (i.e., mandible geometry has weak or no effect on simulated stiffness). Given this observation, we interpret the linearly-scaling mandibular stiffness-volume ratio of *Enhydra* as evidence of a ‘functional release’ of the mandible as a crushing tool by delegating crushing function to the hands. *Enhydra* are known to have dexterous hands that can handle rock tools to pre-process (by smashing) large shelled prey before mastication. The lack of hand tool-use for shell-crushing in *Aonyx* would place the functional demand of crushing large shells entirely on the masticatory system (cranium and mandible). If this interpretation is correct, then the stiff mandible of *Siamogale* suggests this extinct otter likely crushed most or all of its prey using its jaws and did not have the ability to manually process its prey before mastication.

Given the conserved range of ME values across otter species and the significant departure of *Siamogale* in stiffness, we interpret the functional adaptation in *Siamogale* to have occurred by increasing bone strength, rather than by improving the mechanical efficiency of its masticatory system. Such increase in strength, combined with its large size, implies that *Siamogale* was capable of crushing much larger and harder prey than observed in any of the living otter species (roughly six-fold increase in stiffness relative to expectation from linearity). This interpretation is supported by the low stress observed on the *Siamogale* model during bite simulations, especially in the strength of the anterior mandibular corpus and symphyseal regions (Fig. 4). Previous research on rodent ecomorphology suggests that body size alone may be an important axis of ecological diversification and niche partitioning^17^. This may have been the case with *Siamogale*, whose increase in jaw strength appears to be associated only with jaw volume increase and not changes in efficiency. In absence of morphological shape modifications to significantly alter the mechanical efficiency of the masticatory system, the large size of *Siamogale melilutra* would have been critical in allowing the extinct otter to access larger prey in a faunal community where large-bodied predators are essentially unknown in the local fossil record (see below).

The contemporaneous fauna at Shuitangba, where the type specimens of *Siamogale melilutra* were discovered, contains common mammalian species of southeast Asian late Miocene forested habitats (deer, tapir, proboscideans, beavers) as well as aquatics plants such as fox nuts^3^. The abundance of aquatic and near-water environments in that region^18^ may have allowed aquatic carnivorans such as *Siamogale* to become the dominant predators of their ecological communities, outcompeting the larger, more cursorial carnivorans commonly found in more open environments outside of the Shuitangba area. A highly molluscivorous diet was likely for *Siamogale* given the great strength of its jaws, allowing the extinct otters to access foods unavailable to carnivorans without bulbous crushing dentitions (such as felids and ursids, which are known from the same fauna) or are not adapted to living in forested, humid environments (e.g., hyaenids, which are currently not recorded in the Shuitangba fauna)^18^.

In conclusion, our engineering simulation analyses suggest a linear scaling relationship between jaw stiffness and jaw volume in living otters; departure from this linear trend seems to indicate increased mechanical demand for oral processing of hard food items. This linear relationship may be a broader trend among carnivorans not specialized for oral-crushing durophagy. However, extinct carnivorans thought to be specialized in heavily loading their mandibles all exhibit higher mandibular stiffness (lower total strain energy) than expected from isometric model scaling. In particular, the degree of stiffness increase from linear trends observed in extant species is similar between the giant otter *Siamogale* and the marine bear *Kolponomos*. Both *Siamogale* and *Kolponomos* are inferred to have been durophagous molluscivores with emphasis on oral crushing rather than tool-use, suggesting that the acquisition of tool use by living durophagous sea otters functionally released their mandibles from having to deviate from the stiffness-volume isometric model scaling. Low stresses on the mandible during biting also characterize those extinct durophagous molluscivores (Fig. 4g)^16^. Our findings suggest that *Siamogale* does not have a living analog, but exhibits limited similarity to the living oral-crusher *Aonyx* in having significantly stiffer than expected mandibles among otters. Thus, *Siamogale* represents a novel freshwater carnivoran ecomorphology that is lacking in modern ecosystems and exhibits specialization for durophagy by large size and high jaw bone volume instead of increased efficiency.

## Methods

We included 10 of 13 extant otter species in our study. We did not include *Lutra sumatrana* because of its rarity, and a suitable specimen was not available for this study. *Lontra provocax* and *Lutra nippon* were in various publications considered synonymous with *L*. *canadensis* and *L*. *lutra*, respectively^12,19^. Based on morphological and ecological similarity to those respective species, we sampled *L*. *canadensis* and *L*. *lutra* in this study, and not *L*. *provocax* or *L*. *nippon*. The ten species sampled covered the entire range of dietary preferences observed in living Lutrinae (Supplementary Table S1). Computed tomography (CT) scans for the otter species analyzed were obtained from the Institute of Vertebrate Paleontology and Paleoanthropology, Sloan Kettering Cancer Center, American Museum of Natural History, and University Hospitals (for scanning parameters see Supplementary Table S2). Image stacks in DICOM or TIFF formats were imported into Mimics Research version 19 (Materialise, Belgium). The mandibles and crania were highlighted using the bone preset density range as a guide, then delineated manually to highlight all cortical bone. Any remaining trabecular regions were removed during subsequent decimation process (see below), so all models represent cortical bone models only. The segmented images were converted into 3D reconstructions and exported in STL format. All otter species except for *Lontra canadensis* and *Enhydra lutris* were CT-scanned for this study. Models of *L*. *canadensis* and *E*. *lutris* were taken from Tseng *et al*.^16^; in addition, the non-lutrine carnivoran models from the same study were used for comparative analyses.

The 3D reconstructions were then imported into Geomagic Wrap (3D Systems, USA), where the models were decimated to ~200,000 triangular elements, with constrained maximum edge-length ratio of 8, and edge-edge ratio of 6 on all triangles. Cavities representing broken areas or osteological regions not captured during the CT scanning were manually patched using the “fill holes” function. Next, muscle attachment sites for the temporalis, masseter, and medial pterygoid groups were highlighted on both crania and mandibles using bony rugosities and comparative anatomical studies as guides. Because the cranium of *Siamogale melilutra* is badly crushed, only mandibles are simulated. A manual reconstruction of the cranium of *S*. *melilutra* was used to estimate the insertion sites of masticatory muscles on the cranium^3^. Muscle attachment sites and the decimated 3D meshes were exported as binary STL files.

The mandible meshes then were imported into Strand7 (Strand7 Pty Ltd, Sydney, Australia) finite element analysis software. The meshes were cleaned (duplicates nodes removed) and converted into coarse, medium, and fine resolution solid meshes based on the recommendation of Tseng and Flynn^4^. Mandibular muscle insertion sites then were imported, cleaned, and surface areas calculated for input force calculation. Because muscle mass, cross-section area, and activation patterns were not available for the living species analyzed, we estimated muscles as proportional to the attachment area highlighted in each muscle group. The muscle areas (in mm^2^) were multiplied by 0.3 N, and balancing side muscle force were adjusted to 60% of working side muscle force, as in Tseng *et al*.^16^. The MATLAB script BONELOAD was used to distribute the calculated muscle forces over the attachment areas using the tangentially applied option^20^. Muscle centroids were calculated in Geomagic Wrap by calculating the ‘center of gravity’ of cranial muscle attachment sites for each of the left and right temporalis, masseter, and medial pterygoid groups, respectively.

The loaded mandible models were constrained using three nodal restraints - left and right temporomandibular joints (TMJ; at the center of the condylar process) and the unilateral bite point in each simulation scenario^21^. The working side TMJ constraint prevent translational movement in all three axes; the balancing side TMJ constraint allowed translation only in the axis of the TMJ joint. The bite constraint prevent translation only along the long axis of the tooth cusp constrained. Unilateral bites simulated included left and right canines, premolar 2 through 4, and molar 1 to 2 where present. Finally, all models were assigned the same, homogeneous material property approximating mammalian cortical bone, with Young’s Modulus of 18 GPa (gigapascals) and Poisson’s Ratio of 0.3. Because our goal was to assess mechanical response to masticatory forces in the overall mandible, we did not model dental materials separately. Furthermore, the homogeneous models represented dentition and cortical bone only; thin trabecular bones were excluded during the smoothing stage in the Geomagic Wrap program, to prevent over-stiffening the mandible models with trabecular bones being assigned cortical bone material properties. Finite element solutions were calculated using the PCG solver at 10,000 iterations, with an initial tree seed search as a prior. The simulations were conducted on Windows 7 workstations using Strand7. A total of 384 simulations were solved. Model parameters can be found in Supplementary Table S3.

The numerical data extracted from the finite element simulations included (1) nodal reaction forces (“output bite force”) measured at the nodal constraint of each tooth position in the respective bite scenarios, and (2) total strain energy values as calculated from the simulation results. For comparison, nodal reaction force at each tooth locus was scaled by dividing its value by the total input force (sum of working and balancing side temporalis, masseter, and medial pterygoid forces), and termed mechanical efficiency hereafter. The raw, unadjusted total strain energy value for each bite simulation were adjusted relative to the volume and total input force of the *Enhydra lutris* model (chosen arbitrarily as the standard of scaled comparisons) according to the following equation (1)^5^:

$${{\rm{U}}}_{{\rm{B}}\mbox{'}}={({{\rm{V}}}_{{\rm{B}}}{/{\rm{V}}}_{{\rm{A}}})}^{1/3}{({{\rm{F}}}_{{\rm{A}}}{/{\rm{F}}}_{{\rm{B}}})}^{{\rm{2}}}{{\rm{U}}}_{{\rm{B}}}$$

where U_B’_ is the adjusted strain energy value of model B; U_B_ is the unadjusted, raw strain energy values; V is volume, F is total input force, and model A is the comparison model U_B_ is adjusted relatively to.

Linear regression analyses of data were performed in Minitab 17 using the “fit regression model” and “fitted line plot” options to calculate *p-*values of regression coefficients and 95% confidence intervals of regression lines for simulations at all tooth positions. Regression analyses taking phylogeny into account were conducted using the topology and divergence times reconstructed by Koepfli *et al*.^2^. Phylogenetic generalized least squares regression analyses were conducted for the strain energy and volume data using the *ape* package in the R programming environment.

### Data availability

The force-loaded finite element models are uploaded to Dryad at DOI: 10.5061/dryad.ph75c in Strand7 format. Models in other formats (e.g., Nastran) are available upon request to the corresponding author. Raw and scaled simulation output data are included in Supplementary Tables. CT scan data of specimens used in the study are deposited in MorphoSource (http://www.morphosource.org/Detail/ProjectDetail/Show/project_id/343); *Enhydra lutris* and *Lontra canadensis* CT scans are available from a previously published dataset at http://www.morphosource.org/Detail/ProjectDetail/Show/project_id/193.

## Electronic supplementary material

Supplemtnary Tables S1-S6
